# Validation of a novel method for localization of parathyroid adenomas using SPECT/CT

**DOI:** 10.1186/s40463-018-0307-6

**Published:** 2018-10-26

**Authors:** Rachelle A. LeBlanc, Andre Isaac, Jonathan Abele, Vincent L. Biron, David W. J. Côté, Matthew Hearn, Daniel A. O’Connell, Hadi Seikaly, Jeffrey R. Harris

**Affiliations:** 1 Division of Otolaryngology—Head and Neck Surgery, University of Alberta, 1E4 Walter Mackenzie Center, 8440 112 Street, Edmonton, AB T6G 2B7 Canada; 2 Department of Radiology & Diagnostic Imaging, Royal Alexandra Hospital, 1046 Royal Alexandra Hospital – Diagnostic Treatment Center, 2J2.00 WC Mackenzie Health Sciences Centre, 8440 112 Street, Edmonton, AB T6G 2R7 Canada

## Abstract

**Background:**

Accurate localization of parathyroid adenomas is of critical importance in surgical planning for minimally invasive parathyroidectomy. SPECT/CT is considered the investigation of choice but has limitations regarding localization of superior versus inferior adenomas. We proposed a novel method for localization using SPECT/CT by determining the anterior-posterior relationship of the adenoma to a horizontal line in the coronal plane through the tracheoesophageal groove. Our objective was to determine the accuracy, validity, and inter-rater reliability of this method.

**Method:**

This was a retrospective review of patients who underwent parathyroidectomy for a single adenoma between 2010-2017. SPECT/CT images were reviewed by two staff Otolaryngologists, a Radiologist, an Otolaryngology fellow and Otolaryngology resident. Results were compared using intra-operative report as the gold standard.

Overall accuracy in determining superior/inferior and right/left adenomas was calculated, as well as Cohen's Kappa to determine agreement with operative report and inter-rater reliability. The performance was compared to that of the original radiology report.

**Results:**

One hundred thirty patients met criteria and were included. Our method correctly identified the location of the adenoma in terms of both side and superior/inferior position in 80.4% [76 - 84%] of patients, which considerably outperformed the original radiology report at 48.5% [4-78%] accuracy. The agreement level between our method and operative report was high (Kappa=0.717 [0.691-0.743]), as was the inter-rater reliability (Kappa=0.706 [0.674-0.738]).

**Conclusion:**

We report a novel method for localization of parathyroid adenomas using SPECT/CT which outperforms standard radiology reporting. This tool can be used by surgeons and radiologists to better inform and plan for minimally invasive parathyroidectomy.

## Background

Primary hyperparathyroidism (PHPT) is characterized by hypercalcemia and elevated levels of parathyroid hormone (PTH) and is most commonly caused by a single glandular enlargement, or adenoma. Parathyroid adenoma may occur in any of the four glands but tends to involve the inferior glands more commonly than superior glands [1]. The definitive management of primary hyperparathyroidism is surgical.

Although the number and position of parathyroid glands can vary, the most common arrangement involves four glands, two superior and two inferior glands [2]. The superior glands originate embryologically from the fourth pharyngeal pouch, whereas the inferior glands originate from the third pharyngeal pouch. As a result of their embryological migration patterns, the glands tend to have a predictable position with relation to the thyroid, thymus, and recurrent laryngeal nerve. Superior glands tend to be more intimately related to the recurrent laryngeal nerve and lie deep to the plane of the nerve. Inferior glands in contrast tend to lie in a more superficial plane than the nerve and more inferiorly, between the thyroid and thymus [3–6].

Accurate pre-operative localization of parathyroid adenomas is critical for operative planning and the facilitation of minimally invasive surgery. In recent years, there has been development of more effective imaging methods to localize parathyroid adenomas, rather than the previous gold standard of surgical four-gland exploration. Non-invasive preoperative methods for localization of parathyroid adenomas include: ultrasonography, radioiodine or technetium scintigraphy, technetium 99 m sestamibi scintigraphy, computed tomography scan, and magnetic resonance imaging [1, 2, 7, 8]. Recently published studies have shown high utility in the use of single-photon emission CT (SPECT) using sestamibi radionucleotide imaging in combination with CT scanning (SPECT/CT). SPECT/CT has been found to have improved anatomic detail when compared with traditional sestamibi imaging [9].

Determining whether a parathyroid adenoma represents an inferior or superior adenoma can be difficult on imaging. This is preoperative localization is, however, paramount to successful excision, as superior and inferior glands are approached differently during surgery. Inaccurate localization seen in some traditional methods can result in more extensive or revision surgery. We have developed a novel method, known as the Harris method, of localizing parathyroid adenoma as inferior or superior based on SPECT/CT imaging. This method is based on the embryological origin and migration pathway of the parathyroid glands, as opposed to the traditional radiological method of their relation to the thyroid gland only. The technique consists of drawing a horizontal (coronal) line on axial cuts of SPECT/CT, bisecting the tracheoesophageal groove at the level of the cricoid cartilage. Adenomas that are anterior to this line on axial cuts are deemed inferior, while adenomas that are posterior to this line are superior. The aim of our study is to determine the validity and accuracy of this novel method for localization of parathyroid adenomas when compared to traditional radiological reporting, against the gold standard of intra-operative localization.

## Methods

### Study design

Institutional review board approval was obtained from the Human Research Ethics Board at the University of Alberta (Pro00077058). This was a retrospective diagnostic validation study evaluating the use of a novel method of determining the location of a parathyroid adenoma using axial cuts of SPECT/CT.

Participants who underwent parathyroidectomy for a single parathyroid adenoma between November 1, 2010 until November 30, 2017 within the Division of Otolaryngology - Head and Neck Surgery in Edmonton, Alberta were recruited. Eligible patients were those aged > 18 years with a clinical diagnosis of primary hyperparathyroidism. Patients were excluded if they had previous head and neck surgery, greater than one identified parathyroid adenoma, or if radiology reports, SPECT/CT images and/or operative reports were unavailable. Patients were also excluded if there was a failure to identify whether the adenoma was superior or inferior intraoperatively.

Using SPECT/CT images, two staff Otolaryngologist – Head and Neck Surgeons, a Nuclear Medicine Radiologist, an Otolaryngology – Head and Neck Surgery fellow and resident performed the novel method, blinded to the operative findings, on eligible patients that met the inclusion and exclusion criteria. This novel method consisted of finding the level of the cricoid cartilage on an axial cut of the SPECT/CT and drawing a horizontal line bisecting the tracheoesophageal groove. The participants were then to identify whether the adenoma is anterior or posterior to that line. Data was recorded and participants were given immediate feedback as to their diagnostic accuracy. Participants were given 30 scans in one session to prevent fatigue.

The primary outcome was the accuracy of the novel technique of parathyroid adenoma localization based on SPECT/CT using intra-operative identification as the gold standard. The accuracy was compared to that of the original radiology report using cohen’s kappa. Our secondary outcomes were inter-rater reliability of using this novel technique. A clinical diagnosis of PHPT included hypercalcemia and high PTH levels. Operative reports were used to identify the correct location of the adenoma causing hyperparathyroidism. Calcium, PTH, intraoperative identification of the adenoma location, radiology report identification, and the result of the performed task by participants were all recorded.

Statistical analysis was carried out using SPSS software (Statistical Package for the Social Sciences, Version 1.0.0.950). The percentages were calculated by adding the correct answers when compared to the operative report as gold standard. Percentage of each adenoma was recorded and the overall accuracy was the mean of all rater accuracies. Cohen’s kappa was used to determine the inter-rater reliability between the original radiology report and the operative report. Fleiss kappa was used to calculate inter-rater reliability between the raters and the operative report and the raters themselves.

## Results

Three hundred ninety-eight patients were reviewed. 267 patients did not meet inclusion criteria and were excluded. 130 patients met inclusion and exclusion criteria and were included in the study. 104 were female, and 26 were male. The mean age of the patients was 59.3 years. The mean preoperative PTH was 20.2 pmol/L (6.1–81.5 pmol/L) and the mean postoperative PTH was 3.06 pmol/L (0.60–12.40 pmol/L). The mean calcium was calculated to be 2.77 mmol/L (2.37–2.77 mmol/L) (Table 1). 37 patients were identified to have left inferior parathyroid adenomas and 26 patients had left superior parathyroid adenomas. 39 patients had right inferior parathyroid adenomas and 28 patients had right superior parathyroid adenomas (Table 1).

**Table 1:** Patient Demographics

Variable	*N* = 130
Age	59.3 (24–86)
Gender	
Males	26 (20%)
Females	104 (80%)
Preoperative PTH (Average)	20.2 pmol/L (6.1–81.5 pmol/L)
Postoperative PTH (Average)	3.06 pmol/L (.60–12.40 pmol/L)
Preoperative Calcium (Average)	2.77 mmol/L (2.37–2.77 mmol/L)
Parathyroid Location	
Left Inferior	37 (28%)
Left Superior	26 (20%)
Right Inferior	39 (30%)
Right Superior	28 (22%)

**Fig. 1:**
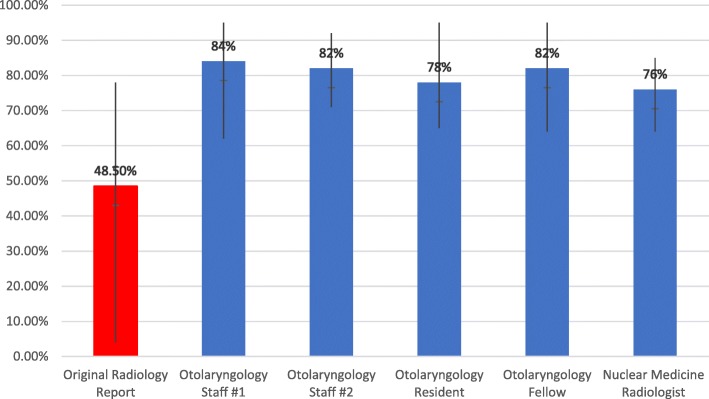
Combined accuracy of correct localization of parathyroid adenomas

**Fig. 2:**
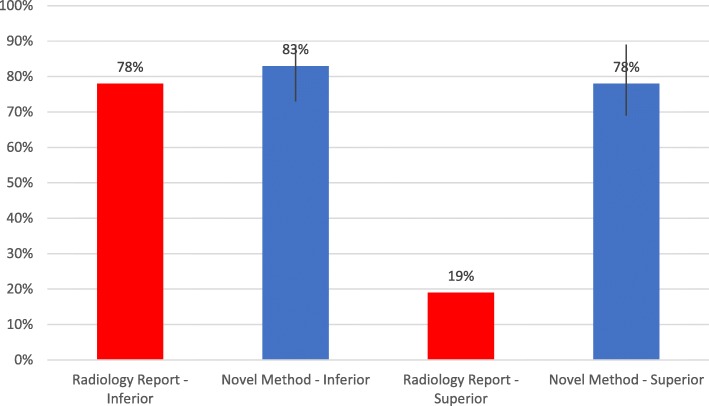
Accuracy of correct localization of Inferior vs Superior parathyroid adenomas

Accuracy was calculated by total correct identified adenoma when compared to the original operative report. The combined accuracy for all raters using the novel method was 80.4% [76–84%]. The accuracy of the original radiology report compared to the operative report was 48.5% [4–78%] (Fig. 1). The accuracy of inferior and superior parathyroid adenomas was also calculated. The overall accuracy of inferior parathyroid adenomas was 83% (73–89%) compared to the original radiology report of 78% (Fig. 2). The overall accuracy for superior parathyroid adenomas was 78% (69–89%) compared to the original radiology report of 19% (Fig. 2).

Reliability was calculated using Cohen’s Kappa and Fleiss Kappa. The original radiology report was compared to the operative report and Cohen’s Kappa was calculated to be 0.468 [0.366–0.570] (Table 2). This is considered to be moderate agreement. When comparing raters to the operative report there was substantial agreement. The Fleiss Kappa was calculated to be 0.717 [0.691–0.743] (Table 3). Overall Fleiss Kappa between raters was calculated and inter-rater reliability was 0.706 [.674–.738] (Table 4). This is considered to be substantial agreement between raters.

**Table 2:** Cohen’s Kappa comparing Operative Report and Original Radiology Report

Weighting	Kappa	Asymptotic Standard Error	Z	Lower 95% Asymptotic CI Bound	Upper 95% Asymptotic CI Bound
Linear	.468	.052	8.891	.366	.570

**Table 3:** Overall Fleiss Kappa for Inter-rater Reliability

Rating Category	Conditional Probability	Kappa	Asymptotic Sandard Error	Z	Lower 95% Asymptotic CI Bound	Upper 95% Asymptotic CI Bound
Inferior	.792	.706	.028	25.441	.651	.760
Superior	.769	.708	.028	25.536	.654	.763

**Table 4:** Fleiss Kappa for Overall Inter-rater Reliability and between Raters and Operative Report

	Kappa	Asymptotic Standard Error	Z	Lower 95% Asymptotic CI Bound	Upper 95% Asymptotic CI Bound
Overall Inter-rater	.706	.016	43.558	.674	.738
Overall Between Raters and Operative Report	.717	.013	54.203	.691	.743

## Discussion

The gold standard of treatment for PHPTH caused by a parathyroid adenoma is surgical resection [10]. When compared to four gland exploration, a minimally invasive approach has been associated with reduced complications such as including injury to the recurrent laryngeal nerve, hypocalcemia and bleeding risks, as well as shorter hospital stay [11–13].

**Fig. 3:**
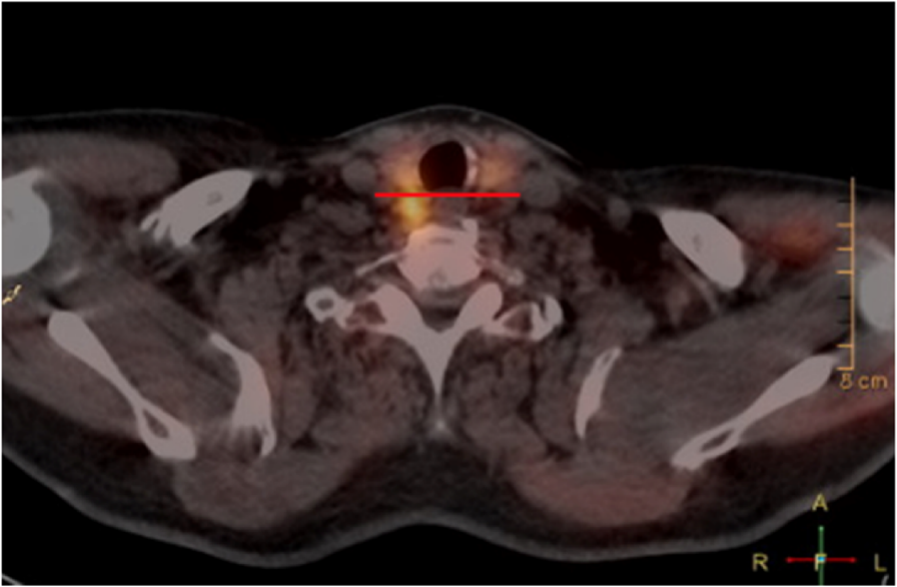
Posterior to the horizontal line on the right deems this to be a right superior parathyroid adenoma

Technological advances have technology has improved accuracy of correct localization of parathyroid adenomas. Preoperatively, it is important to localize these adenomas, as it dictates the surgical approach used to resect the enlarged gland. Of significant clinical importance is the ability to predict the location of the adenoma with respect to the recurrent laryngeal nerve. All superior adenomas (Fig. 3) that would be embryologically and anatomically deep to the recurrent laryngeal nerve require positive identification of the nerve prior to dissection, and resection of, the involved gland. Inferior adenomas are anterior to the recurrent laryngeal nerve and, therefore, dissection on the anterior surface of the gland is safely performed prior to identification of the nerve. Indeed, in some cases the inferior adenoma can be safely excised without identifying the nerve, though in our practice the nerve is identified prior to closure in virtually all cases. Multiple modalities have been used to help achieve localization, however, there is still ambiguity, especially with regards to identifying superior versus inferior gland enlargement.

In 2009, Perrier et al. have developed a nomenclature that provides a precise means of communicating the most frequently encountered parathyroid adenoma locations. Using the Perrier method, a uniform and reliable description of exact locations of parathyroid adenomas can aid in proper localization preoperatively [14]. The system essentially uses the letters A – G to describe exact gland locations. Keidar et al. found that using the Perrier method, 80% of cases accurately localized parathyroid adenomas preoperatively using SPECT/CT [15]. This method however is not widely used in radiology reports due to its complexity.

**Fig. 4:**
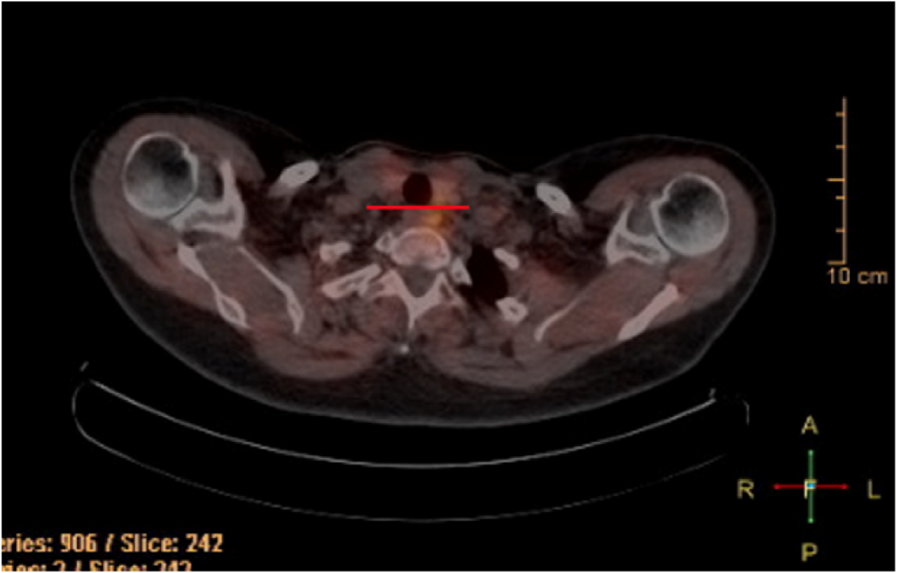
Left superior parathyroid adenoma, as determined by the novel method. This adenoma was reported radiologically as inferior

Instead, radiologists often use the relationship between the parathyroid adenoma and the thyroid gland to decide on its location [16, 17] (Fig. 4). This can be misleading however, as the exact cranio-caudal location with respect to the thyroid gland can vary. Intra-operatively, surgeons more commonly use the relationship to the plane of the recurrent laryngeal nerve in order to localize parathyroid adenomas. Hauty et al., found using technetium-thallium scintiscanning for localizing parathyroid adenomas, a sensitivity in the detection of parathyroid adenomas of 82%, a diagnostic accuracy of 78% and a positive predictive value of 94% [18]. These accuracies are using the thyroid gland as standard of reference. It is important to note that the reason why we see these differences in accuracy is because there is a difference in the standard of reference. The method we have described uses a more embryologically and anatomically sound technique than standard radiological reporting and is thus shown to be more clinically relevant for the surgical approach.

It is important to take into account that the superior glands may never be separated from the thyroid gland during embryological migration [2]. If a superior gland is adenomatous, the potential course could result in the gland in the inferior and posterior position. Dasgupta et al. reported that when radiology reports are duel reported by a surgeon and a nuclear medicine physician, there was a statistically significant improvement in localization [2, 3]. This finding may be attributed to the fact that surgeons may have a better understanding of the anatomy and embryology of the parathyroid glands than the nuclear medicine physician [3, 19]. The use of multiple imaging modalities, our newly described method, and dual reporting with nuclear medicine physicians and surgeons may improve the accuracy of preoperative localization.

The importance of preoperative localization by the operating surgeon cannot be underestimated. Increasing the localization accuracy allows an endocrine surgeon to further minimize the amount of dissection required during surgery which directly reduces the risk profile associated with minimally invasive parathyroid surgery.

There are a few limitations that can be identified in this present study. Using SPECT/CT in itself has limitations including patient motion during the study for accurate registration in SPECT/CT, attenuation and scatter correction, spatial registration, and radiation exposure considerations [20]. Another limitation is the size of the thyroid gland. Theoretically, if the thyroid gland size is larger, it would potentially push the parathyroid glands either more posterior or anterior with respect to the tracheoesophageal groove and could therefore alter our findings or explain some of the incorrect localization results. A larger prospective study may help to address these limitations in the future.

Another limitation is that when the radiology reports were originally dictated, the radiologist did not have the benefit of knowing whether or not there indeed was an adenoma present before reading the study, as opposed to the subjects used for this research study. Thus, localization may have been more challenging for the original radiologist. We attempted to correct for this by eliminating patients that were originally described as non-localizing, regardless of the final operative findings.

We also did not perform intra-rater reliability testing, nor were we able to track a learning curve for our novel method. We plan to collect and analyze these metrics in a follow-up study.

## Conclusion

The Harris method correctly identified the location of the parathyroid adenoma in terms of both side and superior/inferior position in 80.4% of patients, which considerably outperformed the original radiology reports accuracy of 48.5%. There was substantial agreement between our method and the operative report (Kappa = 0.717 [0.691–0.743]), as well as the inter-rater reliability (Kappa = 0.706 [0.674–0.738]). The Harris method is easily performed by the surgeon and radiologist and is less complex and time consuming than the previously reported Perrier method, with very similar localization success. By using this method, preoperative localization of parathyroid adenomas can be improved allowing the surgeon to plan for efficient, effective, and safe minimally invasive parathyroidectomy.

## References

[1] Moreno MA, Callender GG, Woodburn K, Edeiken-Monroe BS, Grubbs EG, Evans DB, Lee JE, Perrier ND (2011). Common locations of parathyroid adenomas. Ann Surg Oncol.

[2] Dasgupta DJ, Navalkisoor S, Ganatra R, Buscome J (2013). The role of single-photon emission computed tomography/ computed tomography in localizing parathyroid adenoma. Nucl Med Commun.

[3] Judson BL, Shaha AR (2008). Nuclear imaging and minimally invasive surgery in the management of hyperparathyroidism. J Nucl Med.

[4] Heffess CS, Wenig B (2008). Embryology, anatomy, and histology. Atlas of head and neck pathology.

[5] Carlson D (2010). Parathyroid pathology: hyperparathyroidism and parathyroid tumors. Arch Pathol Lab Med.

[6] Gray SW, Skandalakis JE, Akin JT (1976). Embryological considerations of thyroid surgery: developmental anatomy of the thyroid, parathyroids and the recurrent laryngeal nerve. Am Surg.

[7] Ciappuccini R, Morera J, Pascal P, Rame JP, Heutte N, Aide N (2012). Dual-phase 99mTc sestamibi scintigraphy with neck and thorax SPECT/CT in primary hyperparathyroidism: a single institution experience. Clin Nucl Med.

[8] Kluijfhout WP, Pasternak JD, Beninato T, Drake FT, Gosnell JE, Shen WT, Duh QY, Allen IE, Vriens MR, de Keizer B, Hope TA, Suh I (2017). Diagnostic performance of computed tomography for parathyroid adenoma localization; a systemic review and meta-analysis. Eur J Radiol.

[9] Hinson AM, Lee DR, Hobbs BA, Fitzgerald RT, Bodenner DL, Stack BC (2015). Preoperative 4D CT localization of nonlocalizing parathyroid adenomas by ultrasound and SPECT-CT. Otolaryngol Head Neck Surg.

[10] Fraser WD (2009). Hyperparathyroidism. Lancet.

[11] Ebner Yaniv, Garti-Gross Yael, Margulis Ariel, Levy Yair, Nabrisky Dan, Ophir Dov, Rotman-Pikielny Pnina (2015). Parathyroid surgery: correlation between pre-operative localization studies and surgical outcomes. Clinical Endocrinology.

[12] Grant CS, Thompson G, Farley D (2005). Primary hyperparathyroidism surgical management since the introduction of minimally invasive parathyroidectomy: Mayo Clinic experience. Arch Surg.

[13] Westerdahl J, Bergenfelz A (2007). Unilateral versus bilateral neck exploration for primary hyperparathyroidism: five-year follow-up of a randomized controlled trial. Ann Surg.

[14] Perrier ND, Edeiken B, Nunez R (2009). A novel nomenclature to classify parathyroid adenomas. World J Surg.

[15] Keidar Z, Solomonov E, Karry R, Frenkel A, Israel O, Mekel M (2017). Preoperative [99mTc] MIBI SPECT/CT interpretation criteria for localization of parathyroid adenomas—correlation with surgical findings. Mol Imaging Biol.

[16] Smith JR, Oates ME (2004). Radionuclide imaging of the parathyroid glands: patterns, pearls, and Ptifalls. Radiographics.

[17] Piciucchi S, Barone D, Gavelli G, Dubini A, Oboldi D, Matteuci F (2012). Primary hyperparathyroidism: imaging to pathology. J Clin Imaging Sci.

[18] Hauty M, Swartz K, McClung M, Lowe DK (1987). Technetium-thallium scintiscanning for localization of parathyroid adenomas and hyperplasia. A reappraisal. Am J Surg.

[19] Melton GB, Somervell H, Friedman KP, Zeiger MA, Civelek AC (2005). Interpretation of 99mTc sestamibi parathyroid SPECT scan is improved when read by the surgeon and nuclear medicine physician together. Nucl Med Commun.

[20] Livieratos L (2015). Technical pitfalls and limitations of SPECT/CT. Semin Nucl Med.

